# Bridging the divide between causal illusions in the laboratory and the real world: the effects of outcome density with a variable continuous outcome

**DOI:** 10.1186/s41235-018-0149-9

**Published:** 2019-01-28

**Authors:** Julie Y. L. Chow, Ben Colagiuri, Evan J. Livesey

**Affiliations:** School of Psychology, University of Sydney, Sydney, NSW 2006 Australia

**Keywords:** illusory causation, contingency learning, causal learning, outcome density

## Abstract

Illusory causation refers to a consistent error in human learning in which the learner develops a false belief that two unrelated events are causally associated. Laboratory studies usually demonstrate illusory causation by presenting two events—a cue (e.g., drug treatment) and a discrete outcome (e.g., patient has recovered from illness)—probabilistically across many trials such that the presence of the cue does not alter the probability of the outcome. Illusory causation in these studies is further augmented when the base rate of the outcome is high, a characteristic known as the outcome density effect. Illusory causation and the outcome density effect provide laboratory models of false beliefs that emerge in everyday life. However, unlike laboratory research, the real-world beliefs to which illusory causation is most applicable (e.g., ineffective health therapies) often involve consequences that are not readily classified in a discrete or binary manner. This study used a causal learning task framed as a medical trial to investigate whether similar outcome density effects emerged when using continuous outcomes. Across two experiments, participants observed outcomes that were either likely to be relatively low (low outcome density) or likely to be relatively high (high outcome density) along a numerical scale from 0 (no health improvement) to 100 (full recovery). In Experiment 1, a bimodal distribution of outcome magnitudes, incorporating variance around a high and low modal value, produced illusory causation and outcome density effects equivalent to a condition with two fixed outcome values. In Experiment 2, the outcome density effect was evident when using unimodal skewed distributions of outcomes that contained more ambiguous values around the midpoint of the scale. Together, these findings provide empirical support for the relevance of the outcome density bias to real-world situations in which outcomes are not binary but occur to differing degrees. This has implications for the way in which we apply our understanding of causal illusions in the laboratory to the development of false beliefs in everyday life.

**Electronic supplementary material:**

The online version of this article (10.1186/s41235-018-0149-9) contains supplementary material, which is available to authorized users.

## Significance

Many of the decisions we make in everyday life are motivated by beliefs about cause and effect. For example, in deciding to use or recommend a medical treatment, patients and health practitioners usually hold a belief that the target treatment will increase the probability that the patient’s health will improve. Critically, laboratory research on contingency learning suggests we are biased to overestimate the causal relationship between a putative cause and an outcome when they are in fact unrelated, particularly when the outcome occurs frequently, a phenomenon known as the outcome density (OD) bias. This effect can account for the prevalent use of ineffective therapies in the treatment of illnesses that have high rates of spontaneous remission, since frequent experiences of symptomatic relief may mislead patients to perceive that the treatment is effective. To date, laboratory experiments investigating these cognitive biases conventionally present the outcome in a discrete fashion (e.g., the patient is sick vs. the patient has recovered). Real-world experiences, on the other hand, are often not binary in nature but have variability and ambiguity, which make inferring causality particularly challenging. The current study is an attempt to bridge the gap between experimental research and real-world experience by investigating the OD bias with continuous and variable outcomes, emulating the need to extract causal relationships from noisy information. This is one step to demonstrating the validity and relevance of contingency learning research in the laboratory to causal learning problems in the real world.

## Background

The ability to extract causal knowledge from direct experience is crucial in helping us make sense of the world. The knowledge that an environmental cue or an action performed by the individual causes a particular outcome can be used to guide our behaviours to maximise desirable outcomes and avoid undesirable ones. For example, if a patient develops a skin rash every time they eat a particular food, the patient may choose to avoid that food in the future to prevent an adverse allergic reaction. Thus, the importance of extracting *accurate* causal beliefs from the available information, including correctly identifying the lack of a causal relationship between unrelated events, is important for our well-being. However, people sometimes identify causality where none exists, and this can affect the way we make judgments and decisions. For these reasons, researchers often take a keen interest in fallacious biases in causal learning. Examples of important real-world phenomena that researchers have argued could be strongly and negatively influenced by learning biases include stereotype formation (Hamilton & Gifford, 1976; Le Pelley et al., 2010), judgment of guilt and voluntariness of confessions in the courtroom (Lassiter, 2002), and the use of potentially ineffective health therapies (Matute, Yarritu, & Vadillo, 2011).

Although the impact of biased causal judgments is relevant to many examples of causal learning in the real world, recent literature has focused on the effects of biased causal learning in medically relevant contexts, particularly beliefs about of the effectiveness of health treatments (e.g., Rottman, Marcum, Thorpe, & Gellad, 2017). This focus is partly motivated by increasing public concern over—and apparent increased use of—pseudo-medicine (Blanco, 2017). In this vein, the rest of this paper will focus on the effects of biased causal learning on judgments about potentially ineffective health treatments. Health treatment choices, like many of the decisions we make in everyday life, are motivated by beliefs about cause and effect. People form a belief about the causal relationship between a putative cause and the outcome (e.g., a drug treatment is thought to improve health) by accumulating evidence from direct experiences with the cause (taking the drug) and outcome events (health improvement). The ability of a putative cause to increase or decrease the probability of an outcome, relative to a base rate in which the cause is not present, is often referred to as *contingency*. Hence, one way in which people form beliefs about cause and effect is through *contingency learning*, acquiring evidence about the likelihood of the outcome after drug treatment and after no treatment (Jenkins & Ward, 1965).

Although people are often accurate when assessing causal relationships via contingency learning (Wasserman, 1990), research has shown that under certain conditions, we are misled to see a causal link between a potential (but ineffective) cause and an outcome (Alloy & Abramson, 1979). In the context of making appropriate decisions about health care, this error in learning is a concern because it may result in the development and maintenance of erroneous beliefs that drive maladaptive choices. For example, despite the lack of support within the scientific community for the efficacy of certain forms of complementary and alternative medicine (CAM), many people still believe in their effectiveness and may even prefer such treatments over those that are scientifically validated (Lilienfeld, Ritschel, Lynn, Cautin, & Latzman, 2014). Indeed, there is now a strong evidence base suggesting that some CAM treatments are completely ineffective (e.g., Barrett et al., 2010). In such cases, the putative cause has no impact on the targeted health outcome and statistical contingency between treatment and outcome is presumably zero or close to it. Nevertheless, frequent personal use of such treatments does not appear to strongly dissuade consumers from purchasing these products.

### Causal learning under zero contingency

Simple contingency learning experiments typically involve two binary events—one potential cause and one outcome—yielding four possible combinations of cause and outcome, as shown in Table 1.

**Table 1:** Contingency matrix showing the four different trial types as a function of whether the cause and outcome are present or absent

	Outcome present	Outcome absent
Cause present	*a*	*b*
Cause absent	*c*	*d*

Manipulations of the covariation between cause and outcome are possible by varying the relative frequency of each trial type, with the resulting contingency conveniently quantified using the ∆p metric (Allan, 1980): 

1 $$ \Delta \mathrm{p}=\mathrm{p}\left(\left.\mathrm{O}\right|\mathrm{C}\right)-\mathrm{p}\left(\mathrm{O}\left|\sim \mathrm{C}\right.\right)=\left[a/\left(a+b\right)\right]-\left[c/\left(c+d\right)\right]. $$

According to this widely used formula, ∆p is positive if the outcome is more likely to occur when the cause is present than when the cause is absent, suggesting that the cause *generates* the outcome. Alternatively, a negative ∆p suggests that the presence of the cause *prevents* the outcome from occurring. For both positive and negative ∆p values, there is some causal relationship between cause and outcome, whereas if the potential cause has no real effect on the outcome, such that p(O|C) = p(O|~C), then ∆p is zero.

Although studies of action–outcome and cue–outcome judgments have generally found that people are sensitive to the strength of positive and negative contingencies (Allan & Jenkins, 1983; Shanks & Dickinson, 1988; Wasserman, 1990), judgments of causation consistently deviate from the ∆p rule when there is no contingency between the potential cause and the outcome (i.e., ∆*p* = 0). The overestimation of causal relationships between two non-contingent events, such that ∆p appears to be either positive or negative when in fact it is zero, is commonly referred to as the illusion of causality (see Matute et al., 2015 for a review). Illusion of causality is an important phenomenon because it represents a consistent error in human learning that is thought to contribute to the development and maintenance of superstitious beliefs and pseudoscientific thinking (Matute et al., 2011).

### Causal illusion and event densities

Many of the studies on illusory causation have explored the probability of the cue and outcome and their effect on generating a false association. Manipulations that increase cause–outcome coincidences (i.e., trial type *a* in Table 1) appear to be particularly effective in producing stronger causal judgments regarding the cue–outcome relationship, regardless of whether the two events are actually causally associated with one another (Blanco, Matute, & Vadillo, 2013; Wasserman, 1990). In other words, a higher coincidence of the cue and outcome results in stronger beliefs that the cue *causes* the outcome. This can be particularly misleading if one of the events has a high rate of occurrence and produces a large number of coincidences for this reason alone.

The OD bias is one example of this effect: it is the tendency to overestimate the relationship between cue and outcome when the outcome occurs frequently. In a classic example, Alloy and Abramson (1979) asked participants to determine the degree of control they possessed over the onset of a green light by pressing a button. In conditions where the button press had absolutely no effect on the light (i.e., zero contingency), participants were more likely to overestimate the action–outcome relationship when the light frequently turned on (high OD) than when it rarely turned on (low OD). This effect has now been replicated across a wide variety of learning tasks with zero-contingency events using binary outcomes (e.g., Matute et al., 2011). Essentially, a high OD increases the frequency of *a* and *c* trials relative to *b* and *d*, even though contingency remains zero, and this is found to be sufficient in generating strong illusions of causality.

Similarly, when there is a high probability of the *cause* occurring (inflating the frequency of *a* and *b* trials relative to *c* and *d*), participants typically report greater causal judgments than when the cause rarely occurs (Allan & Jenkins, 1983; Vadillo, Musca, Blanco, & Matute, 2011). This is known as the cue density effect (e.g., Allan & Jenkins, 1983; Matute et al., 2011; Wasserman, Kao, Van Hamme, Katagiri, & Young, 1996).

### Causal learning about real-world outcomes

Illusory causation is clearly relevant to many of the decisions we must make in day-to-day life. Beliefs about ineffective treatments are grounded in causal illusions, whereby two unrelated events such as consuming *Echinacea* (i.e., an action or cue) and common cold relief (i.e., outcome) are believed to be related in some meaningful way. For example, there is evidence from numerous sources against the efficacy of *Echinacea*  when used to treat the common cold (see Karsch-Völk et al., 2014 for a review), and yet the illusory belief may be persistent among regular consumers who experience *first hand* the zero contingency between *Echinacea* and cold relief. The decisions made based on these false beliefs are incredibly costly: out-of-pocket expenditures on CAM in the United States in 2012 were approximately $30.2 billion (Nahin, Barnes, & Stussman, 2016). Thus, there is a strong imperative to apply cognitive theories of causal learning to real-world problems to formulate ways in which we might mitigate illusory causal beliefs.

The OD effectidentifies a condition that is important for the formation and maintenance of false beliefs. For example, evidence suggests that the strongest beliefs for CAM are for conditions with a high rate of spontaneous remission, which is analogous to having a high OD. In the United States, the use of homeopathic medicines increased by 15% from 2007 to 2012, and in this time they were most commonly used to treat mild respiratory complaints including the common cold, a condition for which intermittent and spontaneous remission is frequent, and the overall probability of recovery is high (Dossett, Davis, Kaptchuk, & Yeh, 2016). The exponential growth of the homeopathic drug market in the United States to an industry worth approximately $3 billion dollars is in conflict with the Food and Drug Administration’s stance that homeopathic products are unproven in their efficacy and potentially dangerous (U.S. Food and Drug Administration, 2017). One potential reason for the persistent use of these unvalidated treatments is the ability for cognitive illusions, including illusory causation, to interfere with the acquisition of evidence-based knowledge. A study by Yarritu, Matute, and Luque (2015) found that participants who developed strong false beliefs about a bogus treatment’s ability to cure a disease in Phase 1 had difficulty learning that a different treatment was actually effective in Phase 2. This suggests that even when patients are given scientifically validated treatment, their ability to accurately recognise improvement in their condition is impaired as a result of previous false beliefs regarding the efficacy of alternative treatments. As a result, they may continue to prefer and seek out treatments that are ineffective in treating their condition.

Although we will not be manipulating the cue density effect in this paper, it is important to note that the cue density effect also has implications for the continued use of ineffective treatments: frequent use may produce stronger causal illusions about the treatment’s effectiveness. This is problematic since most alternative therapies do not produce any side effects. Accordingly, CAM subscribers may be more liberal in using these treatments since the short-term cost of treatment use is low. The effect of the cost of administration was investigated in Blanco, Barberia, and Matute’s (2014) study, in which participants were presented with two fictitious drugs, one with a side effect and one without. The researchers found that participants were more likely to administer the drug without any side effects, and the frequency of drug administration was highly predictive of illusory causation; participants exposed to the drug frequently (i.e., high cue density) were more likely to judge the treatment as being more efficacious than those who rarely administered the drug and thus, had fewer cue-present events (Blanco, Barberia, & Matute, 2014).

The laboratory research on contingency learning and causal illusion thus paints a concerning picture of people’s potential motives for making maladaptive choices. If the principles uncovered in this research are applicable to real-world health beliefs then they suggest that choosing to use ineffective treatments is a self-perpetuating problem: treatments that are used more frequently are perceived to be more effective, and treatments perceived to be effective are used more frequently. This cycle is particularly prevalent for the treatment of ailments with a high rate of spontaneous remission (Blanco et al., 2013).

At face value, experimental evidence for outcome and cue density effects suggest that they provide good laboratory models for how beliefs in ineffective treatments develop. However, one potentially critical difference between the experimental work and their applications is that the former almost exclusively involves situations in which the outcome is binary and invariant (e.g., a green light either does or does not turn on), whereas many real-life outcomes are continuous and noisy. Medical treatments are given in the context of the fluctuating health experiences and biometric data of the patient, which vary in degrees. What might be interpreted as the absence of an outcome (e.g., the patient still presents with the same symptoms and shows no sign of improvement) is not experienced by the individual as the absence of any events. Traditional laboratory-based learning experiments that use health scenarios typically present highly simplified outcomes in a binary and discrete fashion (the patient gets better vs. the patient does not get better). While this aids the task of studying illusory causation, it is not known whether key phenomena (e.g., the OD bias) rely heavily on this simplified way in which events are presented. Continuous and variable outcomes do not fit neatly into the outcome-present vs. outcome-absent dichotomy on which many theories of causal learning are based, and it is unclear whether people readily parse their experiences of continuous noisy events into the presence vs. absence of a target outcome, as in Table 1. This issue was highlighted by Marsh and Ahn (2009) who note that the task of parsing events into the four discrete categories shown in Table 1 is often not a trivial problem, yet is ignored by simple covariation-based models. Indeed, it is less clear whether people are able to (or indeed *need* to) parse their experience of the cue with the outcome into four categorical trial types when the outcomes presented are continuous. Therefore, event information may not come readily classified as evidence for or against the putative cue–outcome relationship.

To our knowledge, no study has previously examined the effects of using more ecologically valid representations of the outcome in examining the OD effect in enhancing the illusion of causality. As such, it is important to test whether continuous, variable, and potentially ambiguous outcomes produce lawful variations in illusory causal judgments in the same way as a simple binary outcome. Thus, we were interested in measuring illusory causation and OD effects using continuous and variable outcomes that are always present to some extent but vary in degree and may be difficult to dichotomise.

### Overview of the current study

The aim of the current study was to test whether illusory causation and OD effects specifically could be generated using an outcome that always occurred but to a varying degree. Our study used a contingency learning task framed as a clinical trial in which participants were presented with a causal scenario and instructed to make judgments about the relationship between a drug cue and a health outcome, in this case, patient recovery. Rather than using binary or discrete outcome events, an outcome was presented on every trial, but its magnitude varied along a continuous scale. To illustrate, instead of the conventional binary outcome where a fictitious patient is either sick or has recovered as a function of treatment or no treatment, patient recovery (i.e., the outcome) was represented on a linear numerical scale from no improvement to full recovery, where the assessment of patient recovery could potentially take on any value within the range.

Participants were presented instructions suggesting a potential causal relationship between the drug cue and health outcome, namely that the drug Cloveritol may increase the recovery rate of patients suffering from a serious illness. Participants then observed a series of trials in which patients were administered Cloveritol or no treatment, with the drug actually having no impact whatsoever on recovery. During training, participants witnessed outcomes presented along a scale from 0 (no improvement) to 100 (full recovery), which they were told represented the patient’s improvement in health. Observed outcomes were sourced from different types of distributions in Experiments 1 and 2. In Experiment 1, we introduced variability in the variable outcome condition by using a bimodal distribution centred at two mean values, a high (80) and low (20) value, and tested whether the presence of variability around these values affected illusory causation and OD effects. The inclusion of variability in the way we represented the outcome was directly contrasted with a fixed-value outcome condition, where low health recovery was represented with a constant value of 20 and high health recovery with a constant value of 80.

**Fig. 1:**
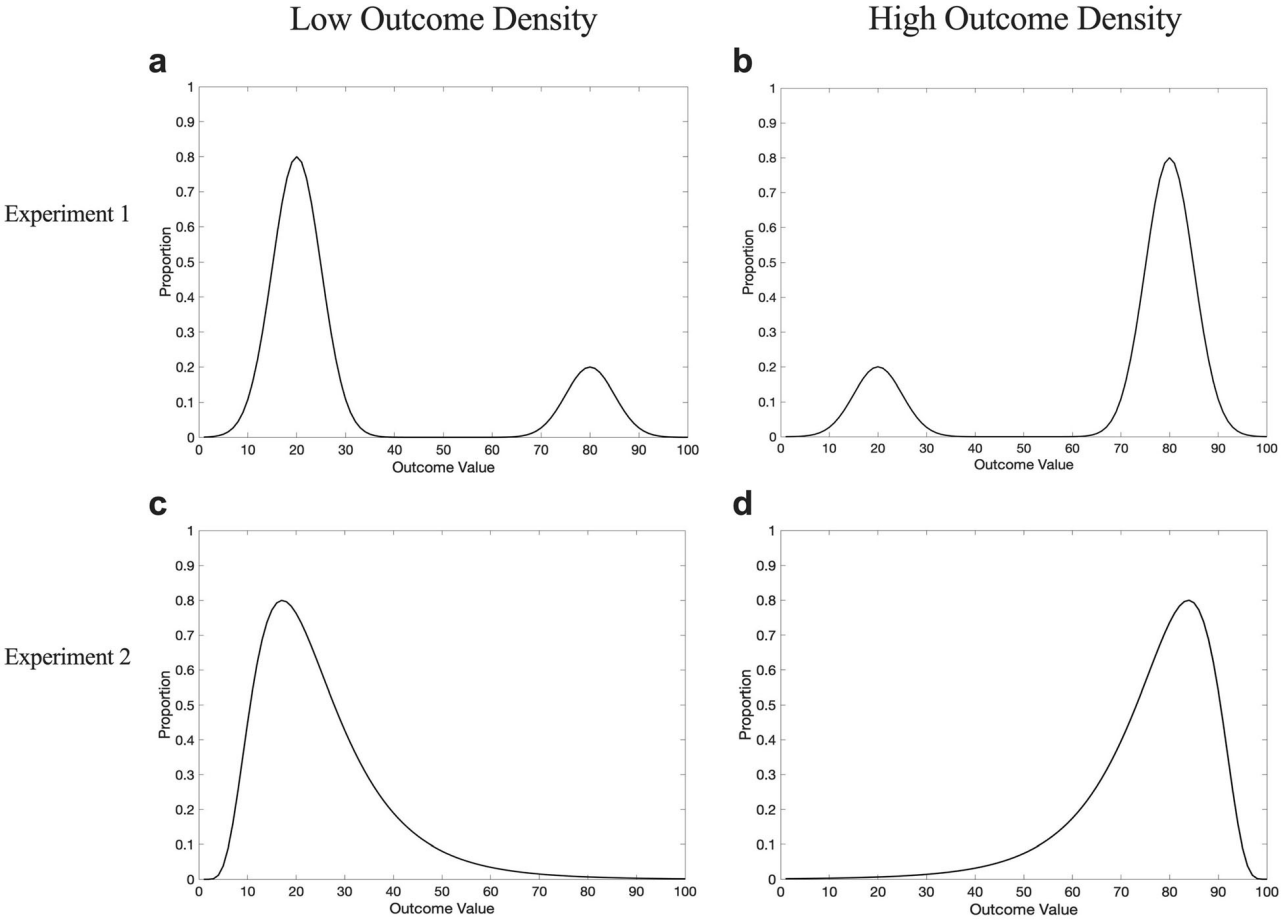
**a** Bimodal outcome distribution presented to participants in variable outcome and low OD condition, where 80% of outcomes were low in magnitude. **b** Bimodal outcome distribution presented to participants in the variable outcome and high OD condition, where 80% of outcomes were high in magnitude. **c** Continuous outcome distribution presented to participants in the low OD condition, where 80% of outcomes were below an outcome value of 50. **d** Continuous outcome distribution presented to participants in the high OD condition, where 80% of outcomes were above an outcome value of 50

In Experiment 2, all participants were presented with continuous and variable outcomes sourced from a unimodal skewed distribution, with either a high or low modal value (see Fig. 1). In both experiments, participants were separated into low OD and high OD conditions, whereby low OD participants observed outcomes that were predominantly low in magnitude with some high magnitude outcomes, whereas high OD participants observed predominantly high magnitude outcomes with some low magnitude outcomes. Although we use the term “outcome density” for consistency with the broader literature, it should be noted that the term entails a slightly different meaning when the outcome is not binary. In our set of experiments, because the outcome occurs on every trial but to a varying degree, a high OD condition is one where a high *magnitude* outcome is more likely to occur than a low magnitude outcome, whereas a low OD condition is when a low magnitude outcome is more likely to occur.

As in most OD effect studies, the critical measure was participants’ causal judgments about the cue, in this case measured using ratings of how effective the drug was in treating the disease.

## Experiment 1

Experiment 1 was conducted to test whether an OD bias produced using a continuous and variable outcome was comparable to that of a fixed-value outcome, most closely resembling the binary outcomes presented in previous research. The OD bias in causal judgments (i.e., ratings of the efficacy of the cause in producing the outcome) is a well-replicated effect but previous studies have used simple and discrete binary outcomes (Alloy & Abramson, 1979; Blanco & Matute, 2015; Langer, 1975). We used a generative causal scenario in which participants were given information suggesting that the drug cue, Cloveritol, may produce faster recovery from illness (i.e., improved health). In Experiment 1, we manipulated OD (low vs. high) and outcome variability (fixed vs. variable) independently as between-subject variables. We used a cue-outcome (i.e., non-instrumental) contingency learning task to keep the probability of the cause (i.e., the cue density) constant across conditions. If differences in outcome variability can sustain similar causal learning biases to those observed with binary outcomes, then the high OD condition should generate greater treatment efficacy ratings than the low OD condition, and this effect should be evident for both the fixed and variable outcome conditions.

### Method

#### Participants

Altogether, 112 participants (78 female, *M*_age_ = 22.2 years, and standard deviation [SD] = 5.35 years) completed the study for class participation or monetary reimbursement. Participants were randomly allocated to one of four experimental conditions according to time of arrival (*n* = 28 in each).

#### Design

The study used a 2 (OD: high vs. low) × 2 (outcome variability: fixed vs. variable) between-subject design. Participants in the fixed outcome condition were presented with an exact-value outcome of 80 or 20 on each trial, presented on a linear scale from 0 to 100. The proportion of each of these two outcomes depended on OD group (80 on 80% of trials in the high OD condition, vs. 80 on 20% of trials in the low OD condition). This condition is analogous to conventional contingency learning paradigms that represent the outcome in a discrete fashion without any variability (i.e., the patient is either sick or has recovered). Participants in the variable outcome condition on the other hand, observed outcomes sampled from a bimodal distribution with outcomes sampled from a low distribution (*M* = 20, SD = 5, and range = 13–27) and a high distribution (*M* = 80, SD = 5, and range = 73–87). The proportion of trials sourced from each distribution depended on OD condition (80% from the high distribution in the high OD condition, vs. 20% from the high distribution for low OD condition). The critical difference between the fixed and variable conditions is, therefore, the presence of variability in outcome values that could be classified as low and high, with the fixed condition experiencing no variability in outcome values (always 20 or 80), and the variable condition experiencing some variability around a low (20) or high (80) outcome value.

All participants completed 100 training trials, 50 with and 50 without the treatment cue. Trials were presented to participants in blocks of 10 such that each block was representative of the total frequency of high and low outcomes in the experiment (Table 2). Drawing on previous research in OD effects, we retain the focus on test judgments of the efficacy of the treatment cue as the primary measure of relevance for the OD effect. However, we also included trial-by-trial predictions of the outcome during training, and an average outcome measure in the test phase. To anticipate the results of both experiments, we found consistent differences across test measures suggesting that causal judgments are particularly conducive to producing OD effects. Discussion of these differences will be saved for the general discussion in Section 5.

**Table 2:** Proportion of total trials (per block and overall) in low and high outcome density conditions. There were 10 blocks of 10 trials matching these proportions, yielding 100 trials in total

	Low density		High density	
	Cloveritol	No treatment	Cloveritol	No treatment
High outcome	0.1	0.1	0.4	0.4
Low outcome	0.4	0.4	0.1	0.1

#### Stimuli and apparatus

**Fig. 2:**
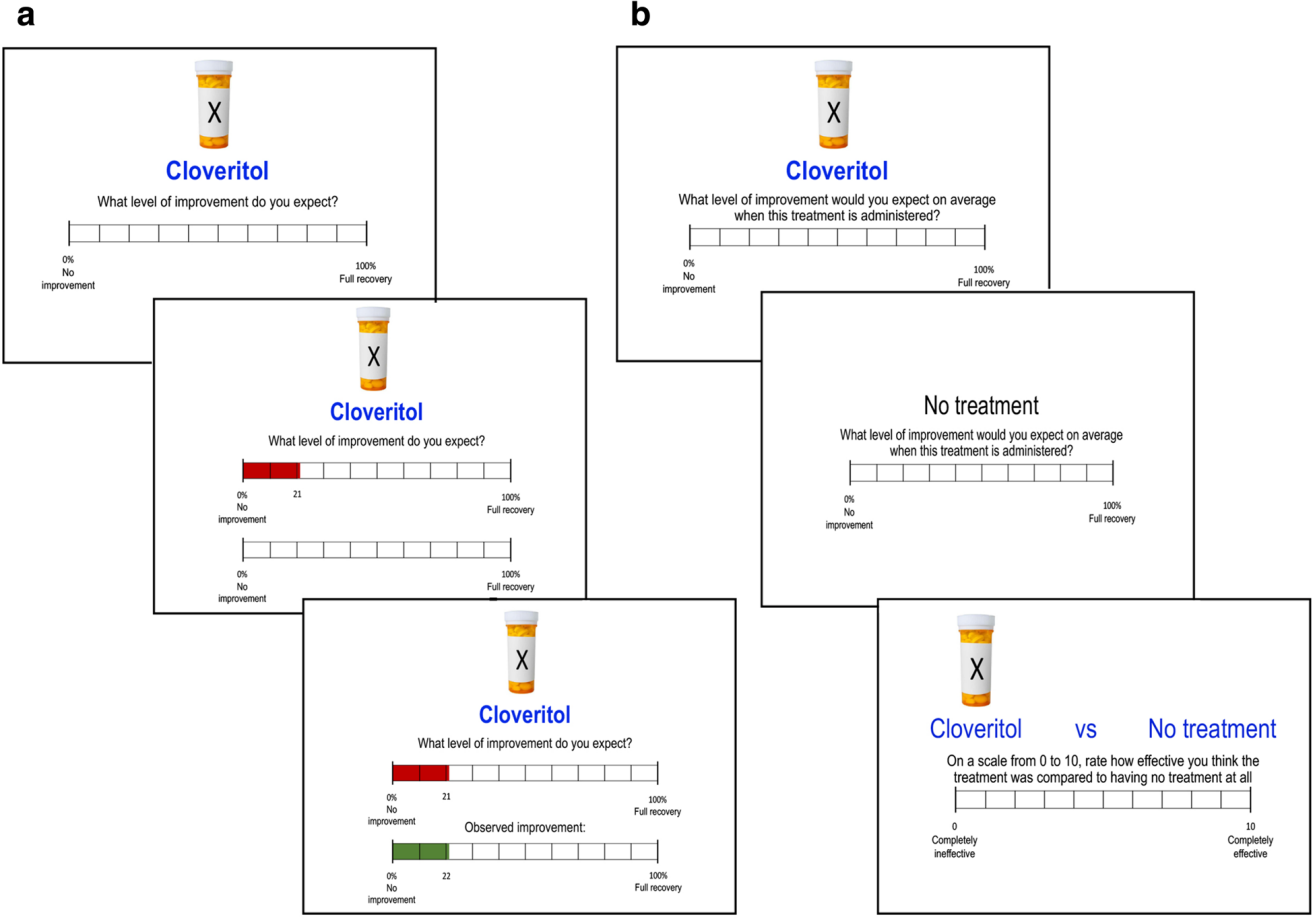
Task schematics for **a** the sequence presented on a single training trial and **b** three test phase questions in Experiments 1 and 2. Causal ratings were made on a scale from 0 to 10 in Experiment 1, and −100 to +100 in Experiment 2

On cue-present trials, administration of the treatment was indicated by presentation of a pill bottle, with the drug name ‘Cloveritol’ written below it (as shown in Fig. 2). On cue-absent trials, ‘no treatment’ appeared without an accompanying image. Predictions in training were made along a visual analogue scale ranging from 0% (no improvement) to 100% (full recovery). The experiment was programmed using MATLAB and the Psychophysics Toolbox extensions (Brainard, 1997; Pelli, 1997).

#### Procedure

Participants were first asked to imagine they were a medical researcher investigating a new illness. Participants were told that a new experimental drug Cloveritol has been created to treat the disease. The objective of the study was for them to test the drug’s efficacy in treating the disease. All participants were told that patients usually take a long time to recover, and a large improvement in health is indicative of a rapid recovery from illness.

During training, participants were presented with trials where they were asked to predict the level of improvement in the patient’s health. Each trial represented a new patient and participants were shown whether the drug or no treatment was administered. Below this cue, a prediction scale was presented and participants were required to predict the patient’s health in that trial by clicking on a point on the scale from 0 (no improvement) to 100 (full recovery). A click on the prediction scale would result in the appearance of a horizontal bar extending from point 0 (extreme left of the scale) to the final click location. The prediction bar was always red, regardless of the magnitude of the prediction. Once a prediction was made and the participant had pressed the space bar to continue, a near-identical scale would appear below with the actual observed health improvement for that trial, animated as a horizontal bar growing from left to right across the scale until it reached the outcome magnitude for that trial. The outcome bar was always green, regardless of the magnitude of the outcome. We used different colours for the two bars to allow participants to differentiate easily between their predictions (red) and the actual observed outcome (green) on that trial.

During the test, participants were instructed to make judgments about the treatment based on their observations during training. Participants were first presented with two average prediction ratings in a randomised order, one each for Cloveritol and no treatment, and were instructed to predict the level of improvement they would expect *on average* if the patient was given Cloveritol or no treatment. Having made average prediction judgments for each cue separately, participants were then presented with the critical causal rating, where both Cloveritol and no treatment cues appeared on the same screen followed by instructions to rate how effective they thought the treatment was relative to no treatment. Ratings were made on a scale from 0 (completely ineffective) to 10 (completely effective). The task procedure is depicted in Fig. 2.

### Results

To recap, previous literature suggests that any effect of OD should be most evident in causal ratings, with greater efficacy ratings reported by participants in the high OD relative to the low OD condition. Importantly for this experiment, if the introduction of variability to the outcome does not produce systematic changes to the OD effect, we would expect the effect of OD to be comparable between the fixed and variable outcome conditions. Given participants were told that the drug should generate greater health improvements, the presence of illusory causation is indexed by greater overall prediction ratings for Cloveritol than no treatment during training and in the test phase. However, previous contingency learning research has often failed to find OD effects in prediction ratings, despite showing the effect in causal judgments (Matute, Vegas, & De Marez, 2002; Shou & Smithson, 2015; Vadillo, Miller, & Matute, 2005).

#### Efficacy ratings at test

**Fig. 3:**
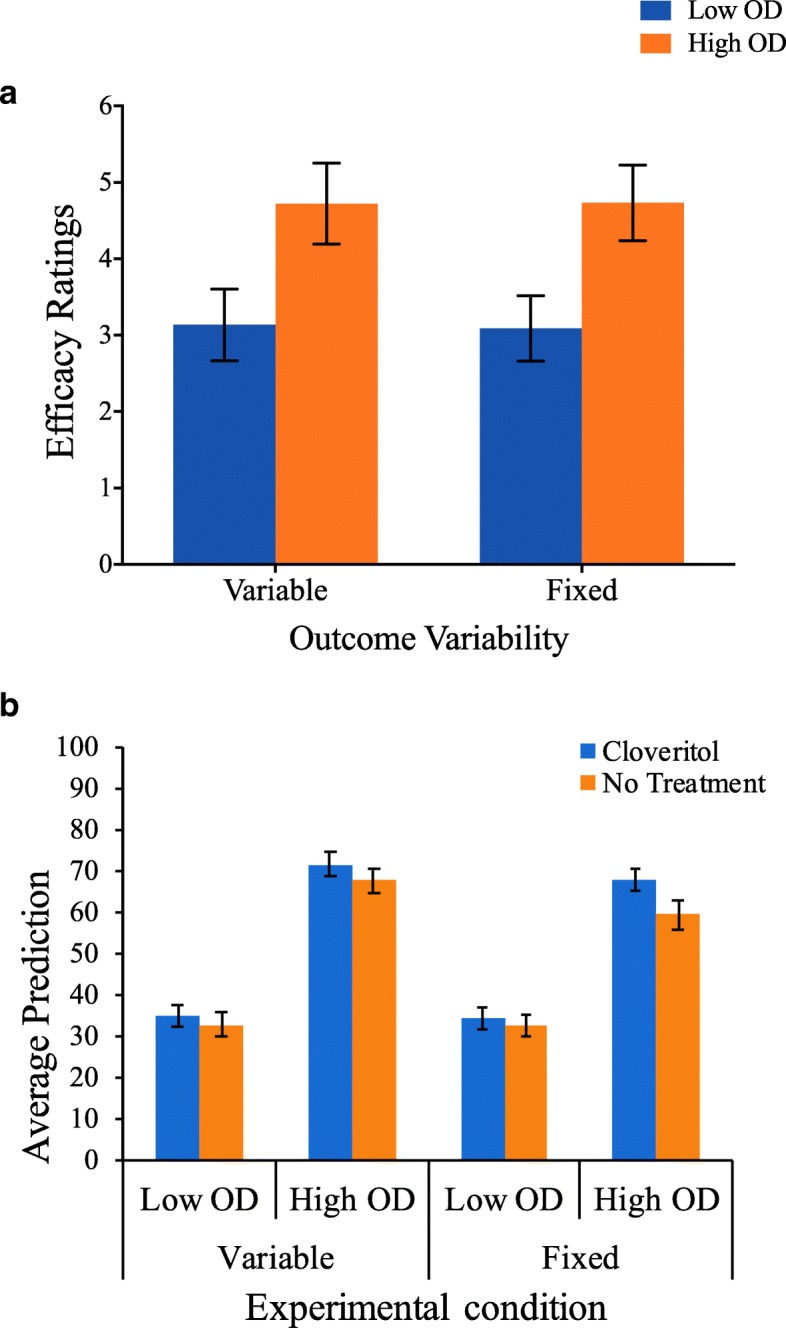
**a** Drug efficacy ratings at test (± standard error) as a function of OD and outcome variability. **b** Average health improvement predictions at test (± standard error) as a function of cue type, OD, and outcome variability. OD outcome density

Treatment efficacy rating, the critical dependent variable, is shown in Fig. 3(a) as a function of OD and outcome variability. We ran a 2 (OD: high vs. low) × 2 (Outcome variability: fixed vs. variable) between-subject ANOVA and found a main effect of OD (*F*(1,108) = 11.3, *p* = .001, and η_p_^2^ = .094), such that participants in the high OD condition (*M* = 4.73 and SD = 2.69) reported significantly greater efficacy ratings than participants in the low OD condition (*M* = 3.11 and SD = 2.34).

Critically, we found no significant interaction effect between OD and outcome variability (*F*(1,108) = .004, *p* = .953, and η_p_^2^ < .001), suggesting that fixed and variable outcomes produce equivalent OD effects. Indeed, significant OD effects were found when participants were presented with variable outcomes (*F*(1,108) = 5.43, *p* = .022, and η_p_^2^ = .048), as well as when they were presented with fixed outcomes (*F*(1,108) = 5.83, *p* = .017, and η_p_^2^ = .051).

#### Average prediction at test

Figure 3(b) illustrates the average prediction of Cloveritol and no treatment across the low and high OD conditions for participants in the fixed and variable outcome variability conditions. A mixed-model ANOVA with OD (high vs. low) and outcome variability (fixed vs. variable) as between-subject factors and cue type (Cloveritol vs. no treatment) as a within-subject factor revealed a main effect of cue type, where mean predictions for Cloveritol (*M* = 52.1 and SD = 22.7) were higher than for no treatment (*M* = 48.1 and SD = 22.1; *F*(1,108) = 6.374, *p* = .013, and η_p_^2^ = .056), suggesting illusory causation was present. However this effect of cue type did not interact with OD (*F*(1,108) = 2.00, *p* = .160, and *η*_*p*_^2^ = .018); that is the difference between Cloveritol and no treatment test trials was not larger for the high OD relative to the low OD condition (such an interaction would indicate an OD effect on this measure). There was also no interaction between cue type and outcome variability (*F <* 1), nor was there a three-way interaction between cue type, outcome variability and OD (*F <* 1).

#### Predictions across training

**Fig. 4:**
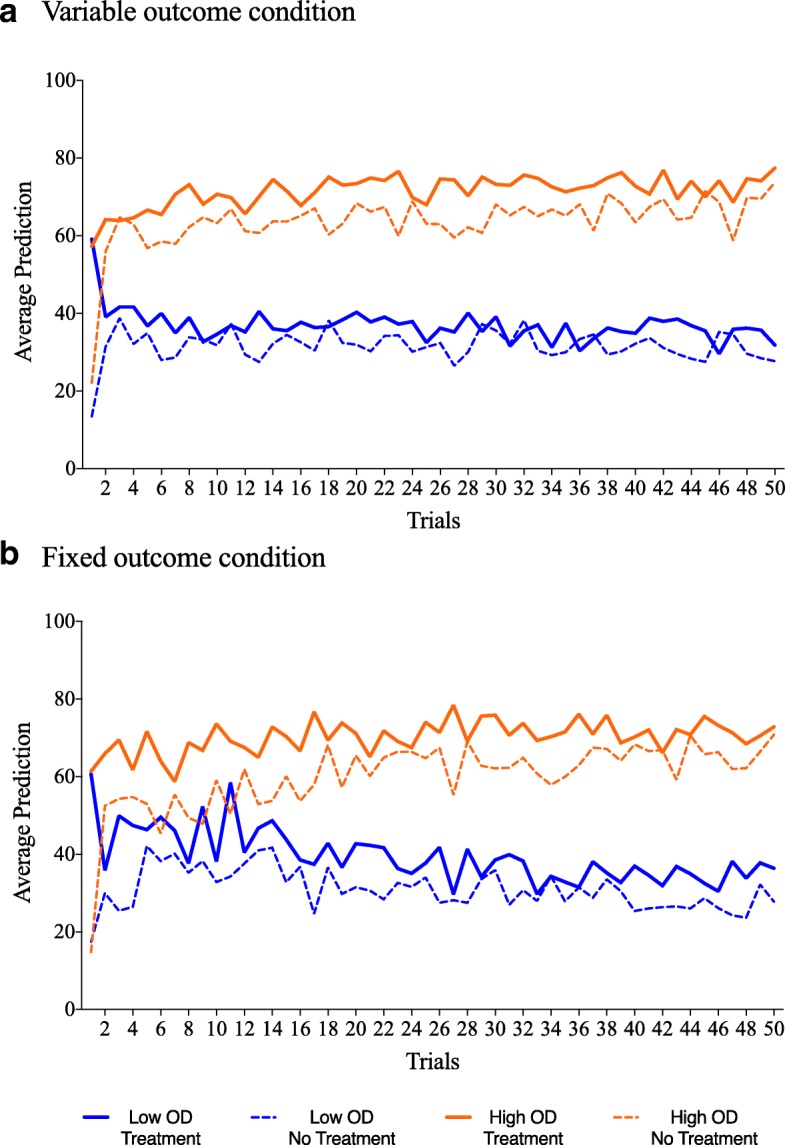
**a** Average predictions during training as a function of OD (low vs. high) and cue type (treatment vs. no treatment) for participants in the variable outcome condition. **b** Average predictions during training as a function of OD and cue type for participants in the fixed outcome condition. OD outcome density

Figure 4 shows mean predictions for the 50 treatment cue trials and 50 no treatment trials during training. The analysis for training predictions was conducted with a mixed-model ANOVA with OD (low vs. high) and outcome variability (fixed vs. variable) as between-subject factors, and cue type (Cloveritol vs. no treatment) and trials (50) as within-subject factors. Like the average predictions  at test, there was a consistent difference in mean prediction for the two cue types, with significantly higher predictions for Cloveritol trials (*M* = 54.6 and SD = 27.1) compared to no treatment trials (*M* = 46.6 and SD = 27.2; *F*(1,108) = 58.5, *p* < .001, and *η*_*p*_^2^ = .351). Again, this effect of cue type did not interact with OD (*F <* 1) or outcome variability (*F*(1,108) = 1.96, *p* = .165, and *η*_*p*_^2^ = .018), nor was there a three-way interaction between these variables (*F <* 1).

### Discussion

Most importantly for our aims, Experiment 1 found a clear OD effect using a variable outcome distribution. The high OD condition produced greater efficacy ratings than the low OD condition and this difference was present in the variable outcome condition, as it was with the fixed outcome condition. Consistent with other studies that have used both prediction judgments and causal judgments (e.g., Allan, Siegel, & Tangen, 2005), we found no evidence of the OD effect in prediction judgments during training and  at test; however, we did find a highly reliable OD effect in the causal ratings. We discuss the lack of OD effects on prediction measures in detail in Section 5, but note that both measures still showed evidence of biased predictions for the treatment cue relative to no treatment (i.e., illusory causation). Outcome predictions were on average higher for treatment cue trials than for no treatment trials, but this bias was no larger for high OD than for low OD conditions. Ratings of the effectiveness of the cue in causing the outcome constitute the most widely used measure of OD in the literature, and we found strong evidence of OD effects on our version of this measure. We, therefore, consider this result to be highly consistent with those of previous studies that have used discrete binary outcomes.

Experiment 1 showed that the use of variable outcomes during training did not significantly alter the results relative to a contingency learning task with fixed-magnitude outcomes, akin to binary outcomes typically presented in these tasks. However, although our bimodal outcome distribution included some variability around a low and high central value, it still lacked ambiguous outcomes (e.g., a value around 50 on a scale of 0–100) and thus, may still be easy to categorise in a binary fashion. For instance, all experienced outcomes could be readily categorised into a low (e.g., a value around 20) and a high magnitude (e.g., a value around 80). The ease with which these outcomes could be dichotomised arguably limits the ecological validity of the experiment. In many real-life situations, the representation of the outcome is considerably more complex and includes both variability and ambiguity and is, thus, more difficult to classify neatly into discrete categories. Experiment 2, therefore, tested for the same OD effects in illusory causation but using a unimodal outcome distribution, in which participants experience a full range of outcome values on a linear scale of 1–99, including intermediate outcomes that are neither low nor high (e.g., they are around the midpoint of the scale).

## Experiment 2

Experiment 2 was mostly identical in procedure to Experiment 1, with changes to the way in which the outcomes presented to participants were distributed. Instead of a bimodal distribution with values centred around 20 and 80, participants experienced outcome values sourced from a single skewed distribution, with values ranging from 1 to 99, but with a modal value that was either high or low. Like Experiment 1, participants in the low OD condition experienced a majority of low magnitude outcomes with some high magnitude outcomes, and this was reversed for participants in the high OD condition. All outcomes presented were independent of the cue. A central difference between the distribution used in Experiment 2 compared to the variable outcome condition of Experiment 1 is the addition of ambiguous outcome values around the mid-range of the scale that are less readily classifiable as low magnitude or high magnitude outcomes. The procedure, thus, arguably has greater ecological validity. We were interested in determining whether we could still obtain OD biases in a contingency learning task with continuous and variable outcomes sampled from a complete range of values, some of which are more decipherable to the participants than others (i.e., intermediate values are more ambiguous given the instructions).

### Method

#### Participants

Altogether, 56 participants (35 female, *M*_age_ = 22.9 years, and SD = 4.44 year) completed the study for a partial course credit or monetary reimbursement. All participants were allocated to one of two experimental conditions according to time of arrival (*n* = 28 in each).

#### Design

The study used a between-subject design with OD (low vs. high OD) as the only manipulation. For the low OD condition, the sample of observed outcomes *O* was positively skewed, created using a truncated ex-Gaussian distribution with a higher proportion of low magnitude outcomes (distribution parameters: μ = 10, σ = 5, τ = 25, and range = 1–99, yielding sample mean = 32 and SD = 20). For the high OD condition, we took the complement of this same distribution (i.e., 100 – *O*) to produce a negatively skewed distribution with a higher proportion of high magnitude outcomes (sample mean = 68 and SD = 20). A sample of values from this distribution was randomly generated but with the further constraint on the proportion of trials with an outcome value below 50: participants in the low OD condition experienced 80% of trials with outcomes below 50, whereas participants in the high OD condition only experienced 20% of trials with outcomes below a value of 50. Ratings of treatment efficacy presented in the test were modified to capture a greater variance in responses, with values ranging from −100 (effectively worsens recovery) to +100 (effectively improves recovery) with a midpoint of 0 (completely ineffective). This modification in Experiment 2 allowed meaningful comparisons to be made between the group means and 0, the midpoint of the scale, whereas 0 represented an extreme end of the scale in Experiment 1. It is also possible that some participants judge that the drug actually makes health improvement less likely (a negative efficacy rating) since the base rate of recovery without the drug was quite high.

All participants received identical causal instructions and the procedure of the study was identical to that of Experiment 1.

### Results and discussion

#### Efficacy ratings at test

**Fig. 5:**
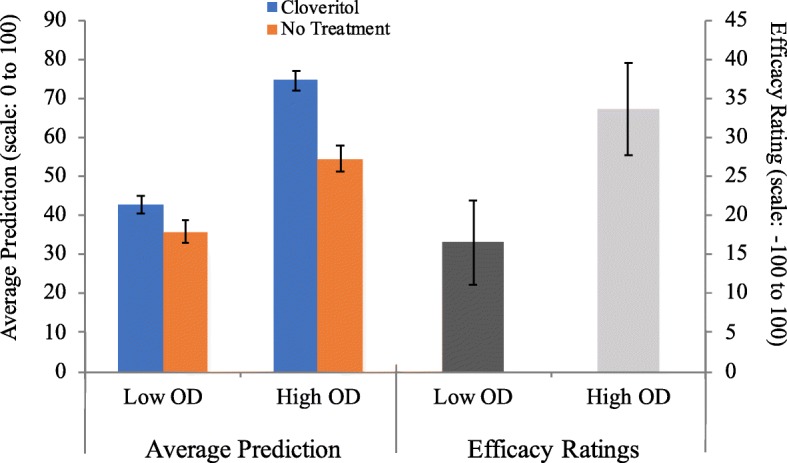
Average prediction for Cloveritol and no treatment at test (± standard error of the mean) (left) and efficacy ratings for treatment relative to no treatment (right) as a function of outcome density. Average predictions were made on a scale from 0 (no improvement) to 100 (full recovery), whereas efficacy ratings were made from a scale of −100 (effectively worsens recovery) to +100 (effectively improves recovery) with a midpoint of 0 (completely ineffective). Negative efficacy ratings indicate that the drug makes patients feel worse, whereas positive values suggest the drug improves patient recovery. OD outcome density

Treatment efficacy rating was the critical dependent variable for observing OD effects; these are shown in Fig. 5 (right). A one-way ANOVA comparing low vs. high OD revealed a main effect of OD (*F*(1,54) = 4.54, *p* = .038, and η_p_^2^ = .078), such that participants in the high OD condition (*M* = 33.6 and SD = 31.7) reported significantly greater efficacy ratings than participants in the low OD condition (*M* = 16.5 and SD = 28.3).

#### Average prediction at test

Figure 5 (left) illustrates the average prediction of Cloveritol and no treatment for participants in low and high OD conditions. Mixed-model ANOVA with OD (low vs. high) as a between-subject factor and cue type (Cloveritol vs. no treatment) as a within-subject factor revealed a similar pattern of results to that in Experiment 1. Mean predictions for Cloveritol (*M* = 56.9 and SD = 17.8) were higher than for no treatment (*M* = 44.8 and SD = 18.0; *F*(1,54) = 24.4, *p* < .001, and η_p_^2^ = .311), indicative of an illusory causation effect. Although the interaction between cue type and OD did not reach significance (*F*(1,54) = 3.06, *p* = .086, and *η*_*p*_^2^ = .054), the marginal *p* value suggests that the difference in prediction ratings for Cloveritol and no treatment was slightly larger in magnitude in the high OD relative to the low OD group, consistent with OD predictions.

#### Predictions across training

**Fig. 6:**
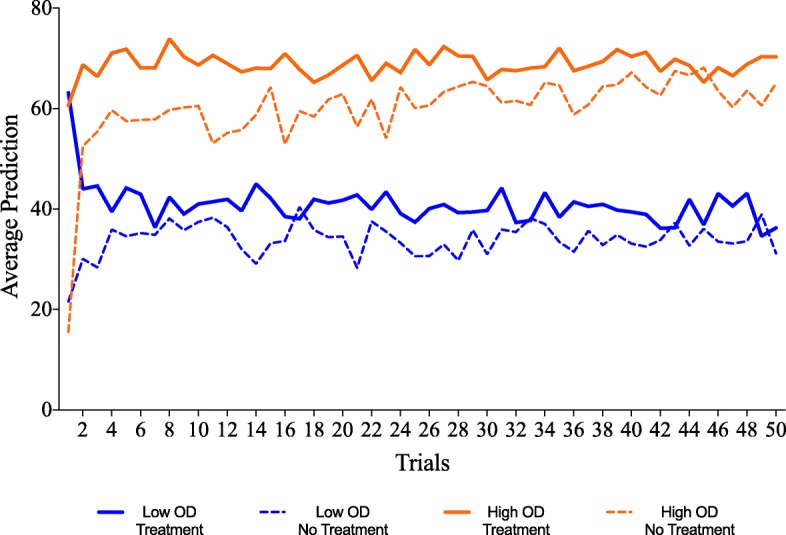
Average health improvement predictions across trials as a function of OD (low vs. high) and cue type (treatment vs. no treatment) for participants in Experiment 2. OD outcome density

Figure 6 shows mean predictions for the 50 treatment cue trials and 50 no treatment trials during training. A mixed-model ANOVA with OD (low vs. high) as a between-subject factor and cue type (Cloveritol vs. no treatment) and trials (50) as a within-subject factor found a consistent difference in mean prediction for the two cue types, with significantly higher predictions in Cloveritol trials (*M* = 53.8 and SD = 24.9) compared to no treatment trials (*M* = 48.9 and SD = 24.5; *F*(1,54) = 24.1, *p* < .001, and *η*_*p*_^2^ = .309). This effect of cue type, which is indicative of an illusory causation effect, was not different for the high OD relative to the low OD condition. Hence, we did not find an OD effect on this measure (*F*(1,54) = .151, *p* = .699, and *η*_*p*_^2^ = .003).

These results are, thus, highly consistent with those of Experiment 1. In this case, we used a skewed unimodal distribution of outcomes in which many trials ended with intermediate consequences that are not as easy to classify as a discrete outcome (e.g., good vs. bad). Nevertheless, we still observed evidence of illusory causation across all measures and an OD effect in causal ratings.

## General discussion

In both experiments we found support for the use of variable outcomes in generating OD effects, with greater efficacy ratings in the high OD relative to the low OD condition. In particular, we found that variability (Experiment 1) and ambiguity (Experiment 2) in the outcomes presented to participants did not alter the systematic biases associated with the OD effect. Importantly, these findings indicate that the OD effect is comparable when using variable outcomes to when using fixed-value outcomes (analogous to discrete events), indicating that OD effects are not confined to simplified binary outcomes. We consistently found an illusory causation effect, indexed by higher prediction ratings for Cloveritol relative to no treatment, and overall positive causal judgments about the efficacy of the treatment relative to no treatment. While researchers have explored causal learning using causes and effects that vary continuously (e.g., Soo & Rottman, 2018), to our knowledge this is the first demonstration of illusory causation and illusory correlation more broadly, using continuous and variable outcomes.

As anticipated, the evidence for OD effects in this study was largely confined to efficacy ratings. While there were consistent differences in the mean predictions for Cloveritol and no treatment during training and  at test, suggestive of illusory causation, these differences were no greater in the high OD group than in the low OD group. Most studies on OD effects have relied only on causal or efficacy ratings  at test as a measure of causal illusion. Indeed, studies that have examined predictions made during training have not always found strong evidence for OD effects, revealing potentially important discrepancies between the decision processes made during training and judgments of causality assessed at the end of training (e.g., Blanco & Matute, 2015; Dèttore & O’Connor, 2013; Waldmann, 2001). Some authors have argued that the dissociation between prediction and causal judgments occurs because these measures are incomparable (Vadillo et al., 2011); predictions during training are typically binary and summed across the training procedure (proportion of “yes” responses to the question *Will the patient recover? Yes/no*), whereas causal judgments are made on a linear numerical scale. That we have observed the same dissociation between causal judgments and predictions made using a linear numerical scale suggests that this argument is insufficient to account for the dissociation. Importantly for our purposes, the discrepancy between prediction and causal judgments is not uncommon in contingency learning literature, suggesting that the current experimental design did not produce unlawful variations in illusory causation effects (Matute et al., 2002; Shou & Smithson, 2015; Vadillo et al., 2005; Vadillo & Matute, 2011).

Our results suggest that illusory causation and the OD effect, in particular, do not depend on processing events in a small number of artificially defined trial types like those illustrated in Table 1. This is important for confirming the validity of contingency learning procedures as laboratory models for the development of causal beliefs in the real world, but it also has theoretical implications. Viable theoretical approaches to causal learning ought to provide a tractable account of illusory correlation effects, explain why they are sensitive to OD, and be applicable to situations involving both discrete and continuous outcomes. Some existing theoretical approaches may be better placed than others to satisfy these constraints. For instance, theorists have shown that associative learning can account for both illusory causation and the OD effect as biases that emerge early in learning and which are corrected over time with greater experience (Matute et al., 2015). This analysis revolves around updating the associative values of the treatment cue and the context by comparing the outcome *prediction* (a continuous value) against a binary teaching signal, usually set to 1 or 0 to represent the presence or absence of a discrete outcome. However, learning algorithms of this kind operate in essentially the same way when continuous values are used for the teaching signal, for instance values that are directly proportional to the magnitude of the outcome. In principle, the same biases early in learning should still be observed in this implementation. It is also possible that other models of causal learning that, in principle, offer explanations for illusory causation and OD effects, such as Griffiths and Tenenbaum’s (2005) causal support theory, can be easily modified for learning about continuous outcomes. However, this remains a goal for future research and theory development.

Notwithstanding the argument above, one might assume that learners have a natural tendency to parse continuous outcome events into discrete outcome categories, and that it is *this* process that gives rise to the OD effect. Participants might spontaneously classify continuous events in discrete terms if doing so enabled them to decide whether outcome information was consistent with or contrary to their current causal hypothesis. This seems relatively plausible for Experiment 1, where the distribution of outcome magnitudes was bimodal and high and low outcomes were distinctly separated, even when variability was introduced to the precise magnitudes observed in each trial. It is less clear that it would be an obvious or indeed useful strategy for participants to adopt in Experiment 2, where the distribution of outcome magnitudes was unimodal. Nevertheless, even in this design, when confronted with ambiguous outcome magnitudes around the middle of the range, it is possible that the learner spontaneously classifies the outcome as being part of a high or low category, in which case they may do so in a way that is biased towards their current causal beliefs (e.g., an outcome around 50 counts as good recovery if Cloveritol was administered but is regarded as poor recovery if no treatment was delivered).

Such a hypothesis parallels previous work with ambiguous *cue* information, which has shown that learners spontaneously categorise ambiguous intermediate observations in a discrete fashion in the direction of their causal hypothesis, and subsequently use them in contingency judgments (Marsh & Ahn, 2009). This hypothesis is speculative and requires further research since the current experiments were not designed to provide evidence that bears on it. However, in any case, we have shown here that, in the presence of variability in a continuous outcome, illusory causation still varies lawfully as a function of OD. We argue that this is an important step because it better emulates the ambiguous and fluctuating properties of real-world experience. Recovery from illness is a pertinent example. We almost always recover from a bout of the common cold, and so the probability of recovery as a long-term outcome is high. However, the rate of recovery and the persistence of unpleasant symptoms during recuperation are highly variable outcomes and are not necessarily easy for the patient or health practitioner to classify in a discrete fashion. Thus, our experimental approach provides a more realistic laboratory model for common day-to-day causal beliefs that impact on decisions we make about health and medical treatments.

An understanding of the role cognitive biases play in influencing how we learn about causal relationships is important as it allows us to recognise the many ways in which we are biased to form erroneous associations between events. Findings from this set of experiments suggest that even when the outcomes are difficult to classify into neat categories, people still show a bias to detect patterns of causality where there is none, and this bias is further inflated by factors that increase the rate of cue–outcome coincidences. As alluded to in the introduction, the overestimation of a causal relationship between unrelated events is particularly problematic when the erroneous belief is used to guide subsequent behaviours. To illustrate, although the efficacy of some CAM treatments, and the “science” behind them, remain highly debated, CAM has evolved to be a commercially successful industry, where users are willing to pay hefty treatment costs without insurance coverage. In Australia, a wide variety of medications are made affordable for the consumer through subsidies provided by the Pharmaceutical Benefits Scheme (PBS). CAM treatments are not routinely funded under the PBS, partly because of their weak evidence base. Even so, consumer expenditure on alternative therapies approached $3.5 billion (Complementary Medicines Australia, 2014), approximately $1.8 billion more than patient expenditure on PBS-funded medications, which approximated $1.5 billion in the same year (Department of Health, 2015). Out-of-pocket expenditure on CAM services is even greater in the United States, with the average user spending approximately $435 a year on complementary health approaches (Nahin et al., 2016). Although some may argue that CAM is at best harmless, research into pseudo-medicine administered to Australian children found that most fatalities related to CAM were a result of the failure to use, or rejection of, conventional medicine in favour of CAM therapies (Lim, Cranswick, & South, 2010). This suggests that illusory causation in the context of health therapies can no longer be perceived as harmless when errors in causal learning may lead to dire consequences. It is, therefore, pertinent that we understand the mechanisms behind these cognitive biases and how we can prevent harmful illusions from developing. To do this, we first need to test these effects in an ecologically valid manner that reflects actual experiences of cue–outcome relationships in the real world.

## Conclusions

In summary, researchers may benefit from adopting a contingency learning paradigm with continuous and variable outcome events that is more ecologically sound, particularly when investigating false causal beliefs in medicine and public health where the consequences of choosing the wrong treatment as a result of biased contingency judgments could have detrimental effects (Freckelton, 2012). This experimental approach may be an important stepping stone to bridging the gap between experimental research and real-world experience.

## Additional file

Additional file 1: Summary file containing deindividualized data, including ratings for all Cloveritol and No treatment trials during training, and ratings at test for Experiments 1 and 2. (XLSX 86 kb)

## Abbreviations

CAM: Complementary and alternative medicine
OD: Outcome density
PBS: Pharmaceutical Benefits Scheme
SD: Standard deviation
